# Supplementary material for: How do traditional media access and mobile phone use affect maternal healthcare service use in Bangladesh? Moderated mediation effects of socioeconomic factors
Source: PLoS One. 2022 Apr 27;17(4):e0266631. doi: 10.1371/journal.pone.0266631 (PMC9045672; doi:10.1371/journal.pone.0266631)
Supplement: Attachment — Submitted filename: PLOS ONE_dr ekong review_260222.docx [file pone.0266631.s002.docx]

**PLOS ONE**

**Peer review**

How do media access and mobile phone use affect maternal healthcare service use in Bangladesh? Moderated mediation effects of socioeconomic factors

**Comments:**

In an attempt to demonstrate the channel complementarity theory, the authors set out to describe the relationship between two channels; media access to health information and mobile technology use for interpersonal healthcare communication among pregnant mothers and utilization of maternal healthcare services. The paper aptly examines the secondary influence of social determinants of healthcare on these channels and consequently maternal health service utilization outcome.

However, the paper has many weaknesses that need to be addressed before it is accepted for publication:

1. **Limitations in dataset analysed:** It appears that the major limitation to the study was the secondary dataset obtained from the Bangladesh Demographic Health Survey which the authors claim did not capture certain information such as the level of internet use and access by the study participants. This has completely eliminated the intractable influence of social media on access to health information in a ‘computer age’. Also, in Page 12, paragraph 2, line 9 and 10, though the authors agree that using more channels of health information might increase utilization of maternal health services, it fails to demonstrate the effect of social media usage on the knowledge, attitude and practices of the study subjects.

It is therefore recommended that since the data source did not capture the datasets that appropriately describe contemporary media channels such as social media and internet usage, the authors may wish to review the title of the paper to reflect the source of data.

2. **Definition of key terms**: While the authors defined ‘have some access’ as ‘Watched TV/listened to radio/read newspapers at least once a week’, it is completely silent on the definition of ‘limited access’. It graded the level of access to media use (some access and limited access) with accompanying degree of maternal health service utilization but failed to do same for mobile phone use assuming that ‘any kind of exposure’ to mobile technology use will positively affect maternal health service utilization outcome. The authors should clearly define ‘limited access’ so as to avoid ambiguity to the target audience.
3. **Oversimplification of findings**: The authors’ address a critical topic in demand creation within a complex system of factors affecting uptake of comprehensive maternal care services amongst pregnant mothers. For instance, the influence of counter moderating factors such as increased distance to health facilities on the use of ANC services were completely ignored. Also, In Page 27, paragraph 2, line 8, the authors ignored the influence of cultural beliefs, practices, and norms on the lack of women’s autonomy in making healthcare decisions for themselves in the developing world by propounding that having a cell phone to make enquiry about nearby health services is a solution.

Therefore, this paper does not sufficiently describe the balance of complex demand and supply forces for achieving desirable maternal health services outcomes.
